# Influence of Inlet Boundary Conditions on the Prediction of Flow Field and Hemolysis in Blood Pumps Using Large-Eddy Simulation

**DOI:** 10.3390/bioengineering10020274

**Published:** 2023-02-20

**Authors:** Wen-Jing Xiang, Jia-Dong Huo, Wei-Tao Wu, Peng Wu

**Affiliations:** 1 Artificial Organ Technology Laboratory, School of Mechanical and Electric Engineering, Soochow University, Suzhou 215000, China; 2 School of Mechanical Engineering, Nanjing University of Science and Technology, Nanjing 210095, China

**Keywords:** blood pump, flow field, hemolysis, inlet boundary condition, turbulent intensity, CFD

## Abstract

Inlet boundary conditions (BC) are one of the uncertainties which may influence the prediction of flow field and hemolysis in blood pumps. This study investigated the influence of inlet BC, including the length of inlet pipe, type of inlet BC (mass flow rate or experimental velocity profile) and turbulent intensity (no perturbation, 5%, 10%, 20%) on the prediction of flow field and hemolysis of a benchmark centrifugal blood pump (the FDA blood pump) and a commercial axial blood pump (Heartmate II), using large-eddy simulation. The results show that the influence of boundary conditions on integral pump performance metrics, including pressure head and hemolysis, is negligible. The influence on local flow structures, such as velocity distributions, mainly existed in the inlet. For the centrifugal FDA blood pump, the influence of type of inlet BC and inlet position on velocity distributions can also be observed at the diffuser. Overall, the effects of position of inlet and type of inlet BC need to be considered if local flow structures are the focus, while the influence of turbulent intensity is negligible and need not be accounted for during numerical simulations of blood pumps.

## 1. Introduction

Blood pumps are increasingly being used in clinical practice as an effective treatment for patients who suffer from heart failure. Computational fluid dynamics (CFD) has been widely used for blood pumps to predict their flow characteristics and mechanical blood damage induced by non-physical stress in blood pumps, as well as optimize and improve the hydraulic properties and blood compatibility of blood pumps, thus shortening the research and development cycle and reducing research and development costs [1,2,3,4,5,6]. The Food and Drug Administration (FDA) of the USA also means to use numerical simulations as a portion of its approval process to conduct a comprehensive evaluation of the safety of medical devices [7]. Nonetheless, because CFD is based on mathematical models for numerical calculations of various physical fields, differences in mathematical models can lead to differences in simulations. This creates uncertainties such as differences in turbulence models, boundary conditions or grid resolution. What’s more, since blood is a shear thinning, viscoelastic fluid, and a concentrated suspension of formed cellular elements (including red blood cells, white blood cells, platelets etc.), the accurate prediction of the hemodynamical field and blood damage has been a challenging task. The accuracy of simulation results has been questioned. As the FDA round robin initiative shows, significant differences were observed not only between CFD results [8,9,10] from different laboratories, but also between velocity fields and hemolysis predicted using CFD results and experimental results [11,12]. Thus, the influence of various uncertainties on the accuracy of CFD prediction of blood pumps should be carefully studied.

Appropriate boundary conditions are important for the convergence and stability of CFD simulations. Inlet boundary condition such as turbulent intensity (TI), velocity profiles and the position of the inlet may have important effects on the flow field and contribute to CFD uncertainties. The influence of inlet boundary conditions on CFD has drawn the attention of CFD researchers in various fields [13,14,15,16]. Cao et al. [17] studied the influence of TI at the inlet of a ventilated closed room on pollutant diffusion and found that TI (2–30%) had an impact on the separation point of inlet jet flow along the upper wall, and that the difference in pollutant concentration could reach 20%. Inlet boundary conditions are crucial for the hemodynamic simulation of cardiovascular systems [18,19,20,21]. Mill et al. [22] explored the effect of different velocity inlets on hemodynamics in the left atrial appendage (LAA). They found that the need for assimilating patient-specific data from medical images into the models, the use of generic pressure waves (rather than constant values) and dynamic left atrium walls achieved more physiological simulation results. Vella et al. [23] investigated the effect of the wall movement of LAA on thrombosis in the atrial appendage. They concluded that the alterations in contractility and morphology associated with atrial fibrillation pathologies play a primary role in establishing hemodynamic conditions which promote higher incidence of ischemic events. In particular, the impairment in contractility determined a decrease in shear strain rate of about 50%, whilst the chamber pathological dilatation contributed to a 30% reduction, indicating increased risk of clot formation. However, the blood flow in the LAA belongs to the transitional flow and laminar flow state, which is the boundary condition study of low Reynolds number, and so not applicable to blood pumps (high Reynolds number). Wu et al. [24] studied the influence of TI on the flow field and hemolysis prediction for the FDA benchmark nozzle model, with turbulence modeled using the Reynold-averaged Navier-Stokes (RANS) method. They found that the location of jet break-down changes with TI. The effect of TI on the flow field and predicted hemolysis is more pronounced with shorter inlets and as high as 38.5%. Nonetheless, the RANS method is known for smoothing out small-scale flow structures. Perturbations imposed at the inlet might be artificially damped out by the RANS method. Large-eddy simulation (LES) is used to directly solve large energy-containing motions and model smaller eddies. Compared with the RANS method, LES can better predict the complex structure of turbulent flow fields and provide more details of flow fields [25,26,27,28].

Nonetheless, the effects of inlet boundary conditions on prediction and hemolysis in blood pumps remains an open question. This paper focuses on the influence of inlet boundary conditions on the prediction of blood pump flow field and hemolysis using LES. The position of the inlet, type of inlet boundary condition (BC) and the TI at the inlet were considered.

## 2. Material and Methods

The influence of inlet BC on downstream hemodynamics should be associated with the downstream flow path. According to the angle between the inlet and outlet pipes, there are two major categories of blood pumps, i.e., centrifugal blood pumps and axial blood pumps. Thus, the centrifugal FDA blood pump and the axial commercial blood pump Heartmate II were employed in this study. The centrifugal FDA blood pump is a benchmark centrifugal blood pump developed by the FDA, with extensive experimental results of flow field and hemolysis at 6 different operating conditions, while Heartmate II is one of the most widely-used blood pumps in clinical practice, with many experimental results as well [10,11,29]. Since this study was a purely numerical one, this will facilitate the validation of the computational results. The computational setup for the simulation of flow field and hemolysis are also included in this section.

### 2.1. Test Case

#### The FDA Blood Pump

**Figure 1:**
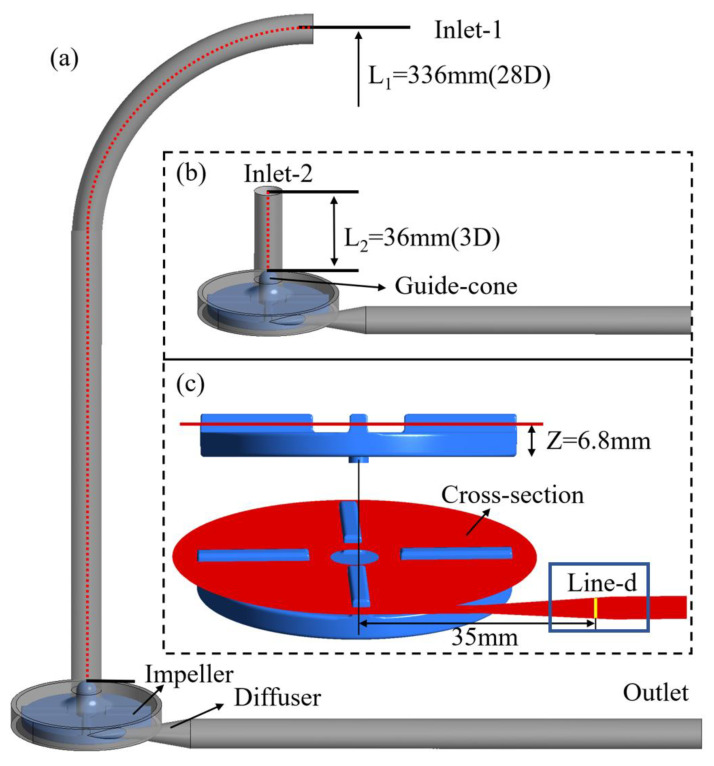
Schematic of the FDA blood pump model: (**a**) Model 1; (**b**) Model 2; (**c**) The experimental data and CFD results are derived from this “cross-section”, with the plane Z = 6.8 mm. The red dotted lines represent the length of the inlet section of Model 1 and Model 2; the yellow line is located at the diffuser of the “cross-section” and 35 mm away from the center of the impeller, which is called Line-d.

The FDA blood pump is a centrifugal pump with a rotor diameter of 52 mm and four rounded blades 3 mm high and 3 mm wide. The diameter D of the inlet section and outlet section of the FDA blood pump is 12 mm. As shown in Figure 1a, the FDA blood pump model was set as Model 1, the position of the inlet was inlet-1, and the length L_1_ of the inlet was 336 mm, i.e., 28D. To study the influence of inlet position, we truncated the inlet section of the FDA blood pump model and set it as Model 2 (as shown in Figure 1b). Its cut-off position was inlet-2, and the length L_2_ of the remaining was 36 mm, i.e., 3D. The red dotted line in the figure was the center line from the position plane of inlet (i.e., inlet-1 and inlet-2) to the top plane of the guide-cone, indicating the length of the inlet section of Model 1 and Model 2, which were 28D and 3D respectively. Model 1 and Model 2 represented the extracorporeal blood pump with external loop pipe and the intracorporal blood pump directly connected to the end of the heart respectively. We extended the outlet section of the two models to 203.5 mm to ensure full blood development. Because of the adverse pressure gradient in the diffuser, the position of “Line-d” (see Figure 1c) is likely to cause flow phenomena such as flow separation and transition. Therefore, the flow here is sensitive to the numerical schemes and turbulence models, and can test the capability of numerical schemes in particular.

PIV tests of the FDA blood pump were carried out [10,11]. The average velocity distributions and pressure heads were tested for six operating conditions, including flow rates from 2.5 L/min to 7 L/min and rotational speeds from 2500 rpm to 3500 rpm. The experimental velocity was taken from 45° of the first quadrant of the impeller and from the line-d of the diffuser in a plane with Z = 6.8 mm (as shown in Figure 1c). The CFD data mentioned in the subsequent results section were also extracted from these lines and compared with the experimental results.

### 2.2. Heartmate II Axial Blood Pump

**Figure 2:**
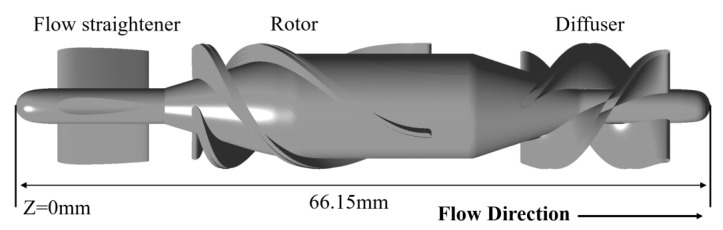
Schematic of Heartmate II.

The HM II pump consists of a flow straightener, an axial impeller, and a diffuser with three blades each (as shown in Figure 2). The front end of the flow straightener is a plane of Z = 0 mm. The total length of the HM II pump is 66.15 mm. The positive direction of the *Z*-axis is consistent with the direction of blood flow. Schüle et al. [29] measured the performance curves of the HM II pump. The pressure head measured at a flow rate of 5 L/min and a rotational speed of 9000 rpm will be used to validate the CFD calculation results.

### 2.3. CFD Analysis

#### CFD Analysis of the FDA Blood Pump

The FDA blood pump was numerically simulated at a flow rate of 6 L/min and a rotational speed of 3500 rpm, roughly the operating point of an extracorporeal blood pump. Under the operating condition, the influence of different inlet boundary conditions on the flow field and hemolysis in the FDA blood pump was explored by changing the position of the inlet, the type of inlet BC and the TI (as shown in Table 1).

Model 1–28D refers to Model 1 as shown in Figure 1a, Model 2–3D refers to Model 2 as shown in Figure 1b. Cases 1–4 were set up to study the effects of the position of the inlet and the type of inlet BC. Experimental velocity profile, measured at the original inlet position (inlet-1, cf. Figure 1), was imposed at the inlet for both models (cases 1 and 4); mass flow rate or uniform velocity profile with the same flow rate as case 1 and 4 was imposed at the inlet for cases 2 and 4. Cases 4–7 were set up to study the effects of TI, with TI (%) being 0, 5, 10 and 20 respectively.

**Figure 3:**
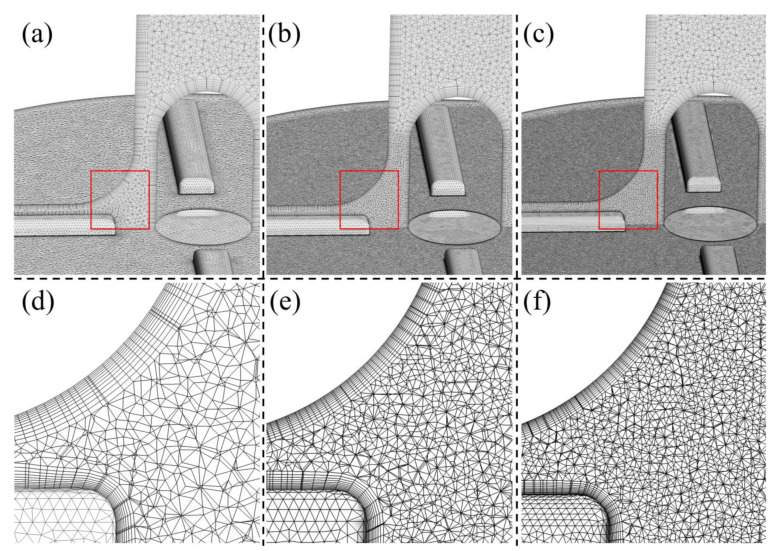
Schematic of the grid of Model 1: (**a**,**d**) 8.26 million grids; (**b**,**e**) 19.50 million grids; (**c**,**f**) 30.90 million grids. (**d**–**f**) show the enlarged details in the red box of (**a**–**c**).

A cylindrical surface was placed downstream of the impeller trailing edge, dividing the pump into a rotating region (around the impeller) and the stationary regions. The fluid domain was preprocessed and meshed into tetrahedral elements, with 11 layers generated to solve the boundary layers using Ansys meshing (Ansys Inc., Canonsburg, PA, USA). Three grids were generated for Model 1, with total elements of 8.26, 19.50, and 30.90 million respectively (see Figure 3). The inlet boundary conditions were set as the same as case 2. Through grid sensitivity analysis, a grid of 19.5 million were identified as suitable grids for the numerical simulation (results are presented in the “Results” section). A grid of 17.4 million was generated for Model 2, with similar settings as Model 1′s 19.5 million grid. The y^+^ was kept within 2 for all grids.

The rotational motion of the rotational regions (impeller) can be approximated using the “sliding-mesh” approach. The blood was regarded as a Newtonian fluid with the density of 1035 kg/m^3^ and the viscosity of 3.5 mPa·s, in line with the experimental data [10,11]. Ansys Fluent (Ansys Inc., Canonsburg, PA, USA) was used to perform the CFD computations. The Semi-implicit Method for Pressure-linked Equation (SIMPLE) method was employed to solve the incompressible N-S equations, which was used to solve both the pressure and velocity coupling in CFD finite volume method. The core of the method is to calculate the pressure field on the basis of the staggered network by using a process of “guess-correction”, so as to achieve the purpose of solving the momentum equation. Turbulence was modeled using the LES WALE model. Time and spatial discretization were bounded second order implicit, and bounded central differencing schemes, respectively. Each impeller rotation was resolved using 960 time steps. The same computational setup was employed and verified in our previous study [30]. The simulations were carried out with a convergence criterion from 10^−3^ to 10^−5^ for the residuals of all equations which drop by 2 magnitudes for each physical time step. After the computational simulations were considered to be converged, time averaging of at least 20 rotor rotations was implemented to gain the average flow field for all cases.

### 2.4. CFD Analysis of HM II

Considering the insufficient pumping capacity of the heart in patients with heart failure, HM II was investigated with an operating condition of 5 L/min and 9000 rpm, with a pressure head of around 60 mm Hg, which is close to the pressure head of an intracorporal blood pump for partial support (70 mm hg). Furthermore, this operating condition was found to be most sensitive to numerical step, more specifically, turbulence models compared with other conditions (cf. [30]). For HM II, only the influence of inlet TI on the flow field and hemolysis was explored (as shown in Table 2). For HM II, a structured hexahedral grid of 5.35 million was generated using Ansys TurboGrid (Ansys Inc., Canonsburg, PA, USA). The y^+^ of the grids was kept within 1.5. The same grid was chosen in [30] through a grid sensitivity analysis. Therefore, the same grid was directly employed in this study.

The time step of HM Ⅱ was 480 time steps per rotation. The blood was regarded as a Newtonian fluid with a density of 1056 kg/m^3^ and a viscosity of 3.5 mPa·s, in line with the experimental data [29]. The other calculation settings, convergence criterion and time averaging method were consistent with the FDA blood pump.

### 2.5. Hemolysis Predictions

Three power-law hemolysis models [31,32,33] were employed in this study to predict hemolysis, which relate hemolysis to effective stress τ and exposure time t through a power-law relationship:

(1) $$HI\left(\%\right)=\frac{hb}{Hb}\times 100=C\tau_{eff}^{\alpha }t^{\beta },$$

where $HI\left(\%\right)$ is the hemolysis index in percentage, $\tau_{eff}$ is the effective stress and a scalar quantity, Hb is the total hemoglobin concentration, hb represents the increase in plasma free hemoglobin; and C, α and β are empirical constants. Three widely used power law models were employed in this study. The effective stress $\tau_{eff}$ is represented in terms of energy dissipation, which can be readily obtained from CFD simulations [34]. The computation of hemolysis was initiated after the statistical convergence of the flow field.

To minimize the uncertainty brought by the three hemolysis models, a variable [35], $HI_{diff}$, expressed the overall change of hemolysis, which was defined as:

(2) $$HI_{diff}=\left(\frac{HI_{GW}^{\prime }}{HI_{GW}}+\frac{HI_{HO}^{\prime }}{HI_{HO}}+\frac{HI_{TZ}^{\prime }}{HI_{TZ}}\right)/3-1,$$

where the denominators represent the predicted hemolysis indices of the cases (case 1, 4, 8 and the “Fine” grid) as comparison, while primes represent the hemolysis indices of the other cases. Subscripts “GW”, “HO” and “TZ” refer to the hemolysis calculated using the three sets of empirical constants [31,32,33].

## 3. Results

### 3.1. Grid Sensitivity Analysis

A grid sensitivity analysis was conducted for Model 1 of the FDA blood pump, and the results are shown in Table 3. For the “Middle” grid, the error of the pressure head and $HI_{diff}$ were less than 1%. As a result, we consider that the cases with the “Middle” grid (19.5 miilion) were sufficiently resolved.

### 3.2. Effects of Inlet BC

#### Results of Flow Field

**Figure 4:**
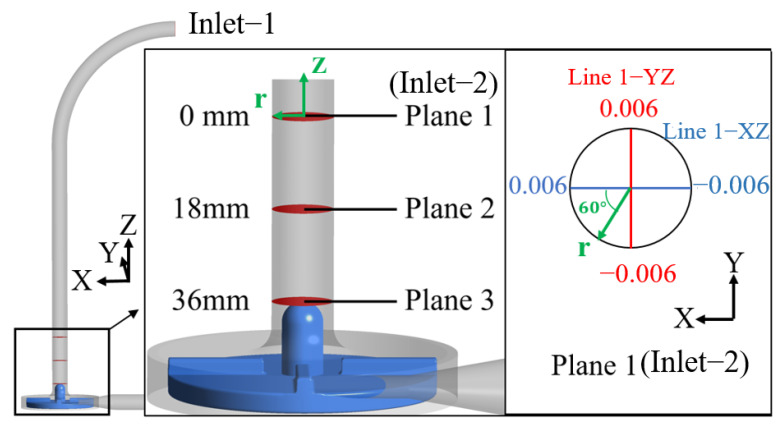
Schematic of the planes which the CFD results were extracted from for the inlet section of the FDA blood pump. The green coordinate is the cylindrical coordinate (r,θ,z), whose Z axis is consistent with the Z axis of the Cartesian coordinate (black). The positive direction of the *X*-axis of the Cartesian coordinate is θ =0° of the cylindrical coordinate.

**Figure 5:**
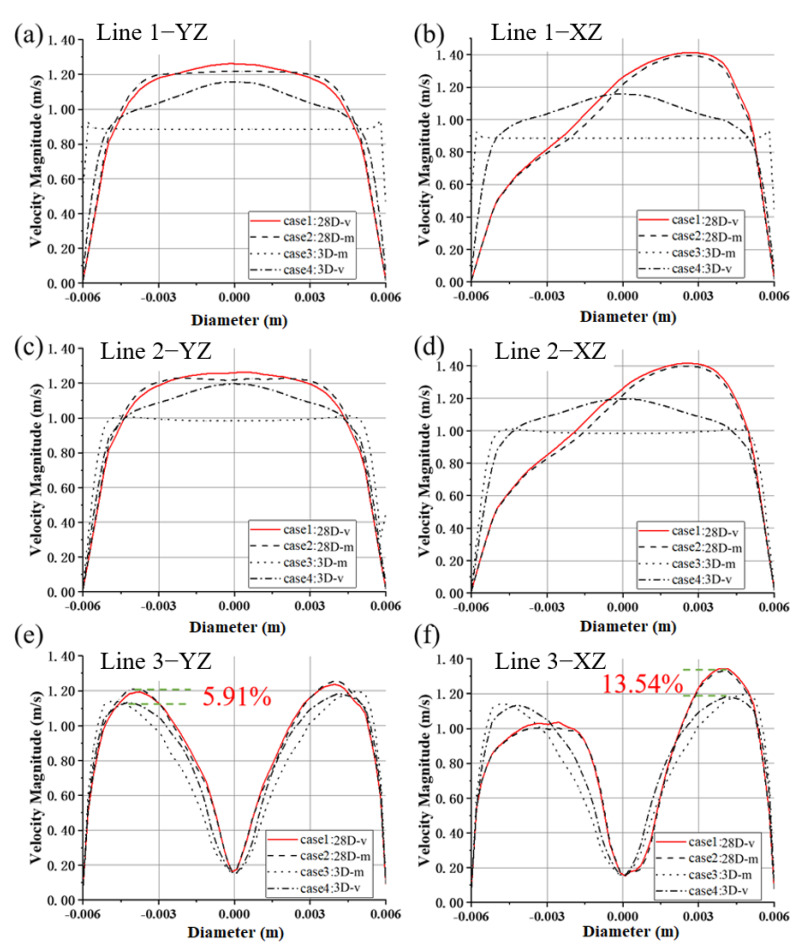
Profiles of velocity magnitude for the FDA blood pump, along the YZ lines of (**a**) plane 1, (**c**) plane 2 and (**e**) plane 3, respectively; along the XZ lines of (**b**) plane 1, (**d**) plane 2 and (**f**) plane 3, respectively.

Figure 4 shows the planes where the CFD results are extracted for the FDA blood pump. Plane 1 is the plane of inlet-2, namely, the inlet plane of Model 2. Plane 2 is 18 mm downstream of plane 1; Plane 3 is 36 mm downstream of plane 1 and is also the top plane of the guide cone. Since there is a bend pipe in the inlet section of Model 1 (the FDA blood pump), the flow field is asymmetric, so velocity profiles averaged over time were plotted at two orthogonal lines (the YZ and XZ lines, see Figure 4) and are shown in Figure 5.

The velocity profile along the YZ line at plane 1 (inlet-2, cf. Figure 5a) is nearly fully developed for Model 1(28D), which is almost the same as that at plane 2 (cf. Figure 5c). For Model 2(3D), the “mass flow inlet” is equivalent to a uniform velocity inlet, as is shown for the velocity profile at plane 1 (inlet-2). At plane 3, flow velocity near the pipe centerline decreases sharply due to the guide cone, and the difference in different velocity profiles still exists (see Figure 5e). The influence of inlet position on velocity peak is up to 5.91%, higher than that of the inlet BC type (1~1.59%).

Along the XZ lines, because of the bend, the peaks of the velocity profile at inlet-2 of Model 1 deviate from the pipe centerline (see Figure 5b,d). The velocity profiles of case 1 and 2 almost match more consistently. At plane 3, the guide cone brings significant change to the flow, and the velocity profiles of different inlet BC types for the same model are very close to each other, while differences can still be observed for the two profiles of experimental velocity profile inlet (case 1 and 4, see Figure 5f). The difference of the velocity peaks is up to 13.54%.

**Figure 6:**
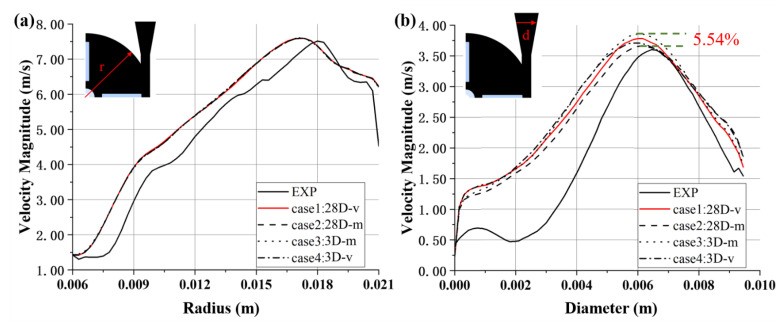
Profiles of velocity magnitude for the FDA blood pump: (**a**) at 45° in the first quadrant; (**b**) along the Line-d. The data were extracted from the plane shown in Figure 1c.

Profiles of velocity magnitude for different inlet BCs of the FDA blood pump are shown in Figure 6. At 45° in the first quadrant of the pump impeller, velocity profiles collapse (see Figure 6a). The velocity peaks are of similar level as experimental results, though the position is offset towards the center. The mean flow field inside the impeller seems to be unaffected by the inlet BC. The differences of predicted velocity profiles across the diffuser are more pronounced, with a maximum difference of 5.54% (between case 2 and case 3, with different positions of inlet). Case 2 (mass flow inlet, and a longer inlet length) is closest to the experimental value. The peak velocities are located at roughly the same position, around 0.006 m, less acentric compared with the experimental peak velocity (see Figure 6b).

#### 3.3. Results of Pressure Head and Hemolysis

Table 4 shows the pressure head values and hemolysis results of FDA blood pumps with different inlet BCs (case 1–4, see Table 1). Under the same operating condition (6 L/min, 3500 rpm), the pressure head of the FDA blood pump measured is 265 mm Hg [30], with a maximum difference of 5.79% (EXP versus case 6). The difference of the pressure head between cases with different inlet lengths (case 1 versus case 4, case 2 versus case 3) is much smaller compared with that between cases with different types of inlet BC (case 1 versus case 2, case 3 versus case 4). For the inlet BC of “mass flow rate”, uniform flows are imposed at the inlet. The development of a uniform flow into a fully developed flow causes a larger pressure drop compared with fully developed flows.

Taking case 1 as a comparison, the $HI_{diff}$ of the other three cases is trivial, all less than 1%, well within the uncertainty of numerical simulations. The computational domain of cases 3 and 4 is slightly smaller than that of cases 1 and 2, with a shorter inlet. This may explain a slightly lower hemolysis for these two cases. Thus, the position and type of inlet have negligible effects on the hemolytic performance of the centrifugal blood pump.

### 3.4. Effects of turbulent intensity

#### Results of Flow Field

**Figure 7:**
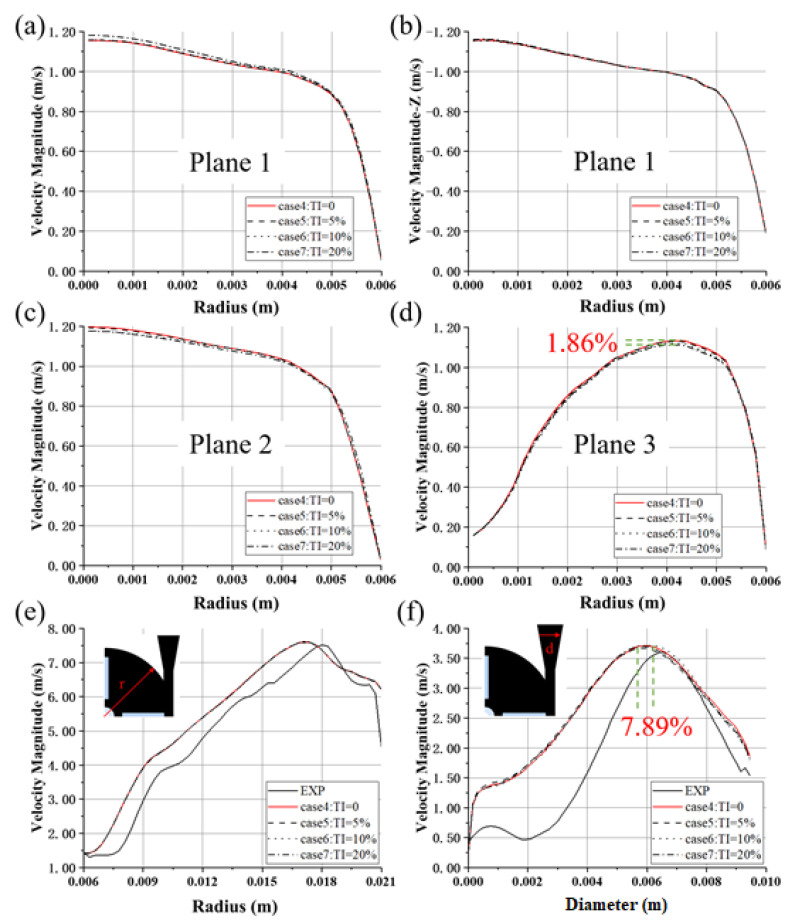
Profiles of velocity magnitude of the FDA blood pump for cases 4–7: (**a**) at plane 1; (**b**) The velocity curve in the Z direction at plane 1; (**c**) at plane 2; (**d**) at plane 3; (**e**) at 45° in the first quadrant; (**f**) at Line-d. The data were averaged both in time and in circumferential directions.

Figure 7 shows the velocities averaged over time and the circumferential direction, along the radial direction at several axial locations. With the increase of TI, the magnitude of the velocity peaks of the inlet-2 (plane 1) also increases, with a maximum difference of 2.23% (between case 4 and case 7), as shown in Figure 7a. Overall, the velocity magnitude increases with the TI at plane 1, but the radial velocities (velocity in the z direction) collapse (see Figure 7b), showing consistent flow rates for all four cases. At plane 2, the velocity profiles become slightly flatter as TI increases and the velocity peak of case 4 (TI = 0) is the largest (cf. Figure 7c). Similar phenomena can also be observed for plane 3 (see Figure 7d), with a maximum difference of 1.86% between the velocity peaks.

The velocity profiles at 45° in the first quadrant of the pump collapse (see Figure 7e). The magnitude of the peaks is of similar level as experimental results, though the position is offset towards the center. The mean flow field inside the impeller seems to be unaffected by the TI. The differences in the position of velocity peaks across the diffuser are more pronounced, with a maximum difference of 7.89% (between cases 5 and 6, with TI = 5% and TI = 10%), while the differences of velocity peaks are less significant, up to 1.10% (between cases 5 and 6) (see Figure 7f).

**Figure 8:**
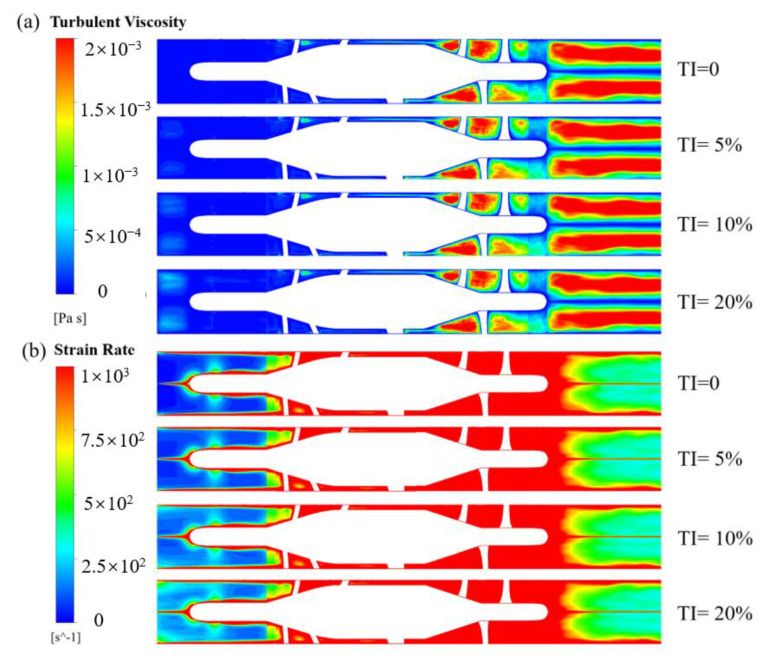
Contour of (**a**) turbulent viscosity and (**b**) strain rate for HM II.

Figure 8 shows the contours of turbulent viscosity and strain rate of HM II. TI has a certain effect on both turbulent viscosity and strain rate in the front end of the flow straightener. With the increase of TI, turbulent viscosity increases as well. However, after the flow entered the rotating region, the influence was attenuated. Similar phenomena were observed for the contours of the strain rate.

**Figure 9:**
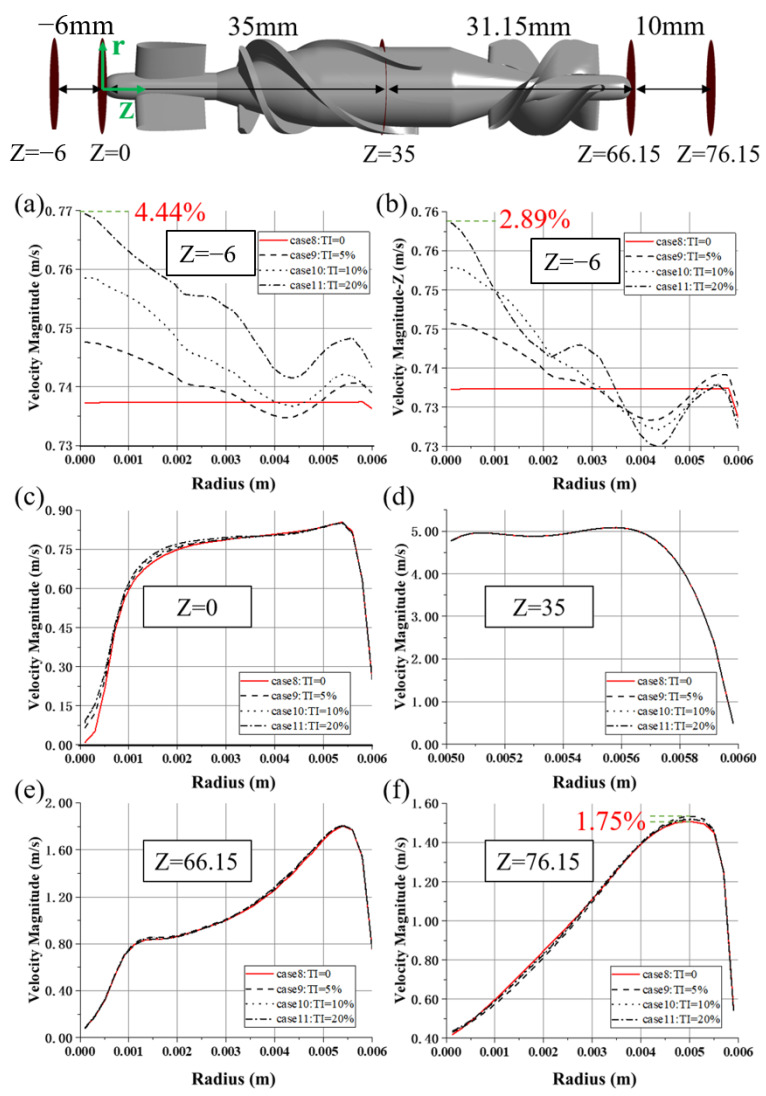
Profiles of two-dimensional velocity magnitude for different TI of HM II: (**a**,**c**–**f**) at the Z = −6, Z = 0, Z = 35, Z = 66.15, Z = 76.15 plane, respectively; (**b**) The velocity curve in the Z direction at the Z = −6 plane. The data were averaged both in time and in circumferential directions. The cylindrical coordinate’s (green) origin is located at the top of the flow straightener of HM II, namely the center of the circle in the z = 0 plane.

Figure 9 shows velocity distributions at several axial locations, averaged both in time and circumferential direction. At the Z = −6 plane, the maximum difference in velocity magnitude is up to 4.44% (between cases 8 and 11, with TI = 0 and TI = 20%, see Figure 9a). Figure 9b shows the distribution of axial velocity along the radial direction at the same plane. Apparently, higher TI caused more disturbance. The difference in velocity distribution diminishes downstream but remains before the flow straightener (see Figure 9c). When blood passes through the region where the rotor is rotating at high speed, the velocity profiles collapse (see Figure 9d,e). Nonetheless, the differences of predicted velocity profiles at the z = 76.15 plane (see Figure 9f) reemerged, with a maximum difference of 1.75% (between cases 8 and 9, with TI = 0 and TI = 5%).

### 3.5. Results of Pressure Head and Hemolysis

Table 5 shows the influence of TI on pressure head and hemolysis. Under the same operating condition (5 L/min, 9000 rpm), the pressure head of HM II as measured was 53 mm Hg [29,30], with the maximum difference of −3.77% (EXP versus case 11). It can be seen that the effect of TI on the FDA blood pump and HM II head was almost negligible. Cases 4 and 8 were used as a baseline when computing $HI_{diff}$. When TI varied, the $HI_{diff}$ was within 2% for the FDA blood pump, and within 0.5% for the HM2, which was within the uncertainty of hemolysis predictions. The TI (0~20%) has little influence on the hemolysis performance of blood pumps. In the computational setup for hemolysis simulation, its effect can be ignored.

## 4. Discussion

Inlet boundary conditions are one of the uncertainties of CFD, which may influence the prediction of the hydrodynamic and hemolytic performance of blood pumps. Studies on the effect of inlet boundary conditions on the prediction of blood pumps are still lacking.

In this study, the working scenarios of an intracorporal blood pump and an extracorporeal blood pump were fully considered, and two blood pump models with inlet length of 28D and 3D were set (Model 1 and Model 2). Furthermore, the effects of types of inlet BC on the hydrodynamic and hemolytic performance of the two models were considered. In addition, the effects of TI (0, 5%, 10%, 20%) on centrifugal and axial blood pumps were also considered. To improve the accuracy of CFD calculation, LES was employed to calculate the flow field of the blood pump in detail. We found that the influence of boundary conditions on integral pump performance metrics, including pressure head and hemolysis, was negligible. However, the influence on local flow structures, such as velocity distribution, were different, and the main difference existed in the inlet (see Figure 5, Figure 6, Figure 7, Figure 8 and Figure 9). The difference in mean velocity distributions at the impeller almost disappeared. The reason might be that high-speed rotations of the impeller attenuate the velocity difference at the inlet. Nonetheless, differences in velocity distributions emerged again at the diffuser (see Figure 6b, Figure 7f and Figure 9f). Higher-order turbulence statistics at the impeller and the diffuser were collected and compared, and differences were observed at the impeller. This might lead to the velocity differences at the diffuser. Nevertheless, there is no obvious correlation between the differences of higher-order statistics in the impeller and the differences of the velocity distribution in the diffuser.

The effects of inlet BCs on the hydrodynamic and hemolytic performance of blood pumps were also investigated in this study. In a previous study, Wu et al. [24] investigated the influence of inlet TI on the prediction of hemolysis in the FDA benchmark nozzle model. They found that for the FDA nozzle model, the influence of TI on the prediction of hemolysis is small, while for the truncated FDA nozzle model, the influence of TI is remarkable. That is, TI has little influence on the overall performance of the FDA nozzle model, but it still has influence on the local flow field (inlet). However, although Wu et al. [24] considered the effects of TI on the hemolysis performance of blood contact devices, they did not consider the effects of complex geometric structures such as blood pumps and complex working conditions such as high-speed rotations. We investigated the influence of inlet BCs, including but not limited to TI, for blood pumps. On the other hand, the RANS method was employed in [24], which is very dissipative and less sensitive to computational setup [25,26,27,30,36]. Therefore, the influence of inlet BCs is not as pronounced as the scenario of LES.

This study also had some limitations. Firstly, only one working condition was considered for each pump in this study. Nonetheless, although two working conditions were different pump type and speed, the influence of TI was similar and negligible. Thus, we believe the findings concerning the influence of inlet BC are qualitative. On the other hand, the TI at the inlet was prescribed in the form of random white noise, without correlations in time and space. For a more comprehensive study of inlet BC, further physical inlet BC will be considered in our future work.

## 5. Conclusions

This study investigated the influence of different inlet boundary conditions on the flow field and hemolysis performance of blood pumps. The influence of inlet position, type of inlet BC and inlet TI were investigated. The influence of pump inlet length on integral pump performance metrics, such as pressure head and hemolysis, is negligible. On the other hand, its influence on local flow structures such as velocity distributions do exist, and mainly exist at the inlet and diffuser. It also can be concluded that the different inlet positions and types of inlet BC have negligible influence on the velocity fields at the impeller due to the high-speed rotations of the impeller. Concerning the influence of inlet BC type, the inlet of the velocity profile (cases 1 and 4) induced a larger pressure drop at the inlet (3–4 mm Hg higher) compared with the inlet of mass flow rate (cases 2 and 3). This pressure drop was also higher than the pressure drop between inlet-1 and inlet-2 (around 1 mm Hg). Thus, for a most accurate simulation of a centrifugal blood pump, we recommend extending the inlet section and imposing the inlet’s velocity profile to allow the flow to get fully developed. When computing power is limited, a shorter inlet section and experimental velocity profile inlet can be chosen to save time. However, for certain scenarios, such as intracorporal blood pumps, where blood enters the pump inlet cannula directly from the ventricle, a further extension of the inlet and velocity inlet BC are neither necessary nor in line with the actual situation.

The influence of TI mainly exists in the inlet section of the two blood pumps. After blood entered the rotating region, the circumferential velocity increased rapidly due to impeller rotation. The influence of inlet TI on velocity distributions diminished or even disappeared in the rotating region as well as the downstream regions. The influence on the pressure head and hemolysis of both blood pumps was also negligible. Thus, the conclusion is that the influence of TI is attenuated by high-speed rotations of the impeller. The effects of TI need not be accounted for during numerical simulations of blood pumps.

This conclusion is important for determining appropriate inlet BCs of blood pumps, improving the accuracy of numerical simulations, as well as guiding the design and optimization of blood pumps.

## Figures and Tables

**Table 1:** Cases for the FDA blood pump with different inlet boundary conditions.

Case	Model	Type of Inlet BC	TI (%)
1	1–28D	Experimental velocity profile	No perturbation
2	1–28D	Mass flow rate	No perturbation
3	2–3D	Mass flow rate	No perturbation
4	2–3D	Experimental velocity profile	No perturbation
5	2–3D	Experimental velocity profile	5
6	2–3D	Experimental velocity profile	10
7	2–3D	Experimental velocity profile	20

**Table 2:** Different inlet TI for HM Ⅱ.

Case	Model	Type of Inlet BC	TI (%)
8	HM Ⅱ	Mass flow rate	No perturbation
9			5
10			10
11			20

**Table 3:** Results of grid sensitivity analysis.

Mesh	$\mathrm{Cells}\;(\times 10^{6})$	P (mm Hg)	Error of P (%)	$HI_{diff}$ (%)
Coarse	8.26	274.63	−1.46	−1.92
Middle	19.50	277.67	−0.37	−0.54
Fine	30.90	278.69	/	/

P: predicted pressure head. Error of P (%), defined as $\left|P-P_{0}\right|/P_{0}$, where $P_{0}$ is the pressure head predicted with the fine mesh.

**Table 4:** Results of pressure head and hemolysis.

Case	1	2	3	4
Pressure Head (mm Hg)	280.34	277.67	276.75	280.01
$HI_{diff}$ (%)	/	−0.19	−0.32	−0.91

Pressure head and hemolysis values were calculated from inlet-2.

**Table 5:** Results of pressure head and hemolysis.

Case	4	5	6	7	8	9	10	11
Pressure Head (mm Hg)	280.01	279.73	281.77	279.54	52.67	52.24	52.11	51.69
$HI_{diff}$ (%)	/	1.60	1.51	1.21	/	−0.42	−0.19	0.38

## Data Availability

The study does not report any data.
